# Enzyme–Metabolite Network Analysis of Endometrial Cancer-Derived Extracellular Vesicles Through Integrated Proteomics and Metabolomics

**DOI:** 10.3390/ijms27177703

**Published:** 2026-08-28

**Authors:** Giovanni Di Lorenzo, Feras Kharrat, Fabio Hollan, Valeria Capaci, Nour Balasan, Michelangelo Aloisio, Federica Caponnetto, Antonio Paolo Beltrami, Federico Romano, Giuseppe Ricci, Blendi Ura

**Affiliations:** 1 Institute for Maternal and Child Health, IRCCS ‘Burlo Garofolo’, 65/1 Via dell’Istria, 34137 Trieste, Italy; giovanni.dilorenzo@burlo.trieste.it (G.D.L.); feras.kharrat@burlo.trieste.it (F.K.); nour.balasan@burlo.trieste.it (N.B.); michelangelo.aloisio@burlo.trieste.it (M.A.); federico.romano@burlo.trieste.it (F.R.); giuseppe.ricci@burlo.trieste.it (G.R.); blendi.ura@burlo.trieste.it (B.U.); 2 Department of Chemical and Pharmaceutical Sciences, Institution University of Trieste, Via L. Giorgieri 1, 34127 Trieste, Italy; hollan@units.it; 3 Department of Medicine, University of Udine, 33100 Udine, Italy; federica.caponnetto@uniud.it (F.C.); antonio.beltrami@uniud.it (A.P.B.); 4 Azienda Sanitaria Universitaria Friuli Centrale, 33100 Udine, Italy; 5 Department of Medicine, Surgery and Health Sciences, University of Trieste, 34149 Trieste, Italy

**Keywords:** metabolomics, proteomics, extracellular vesicles, data independence acquisition, multi-omics

## Abstract

Endometrial cancer (EC) is the most common gynecological malignancy in high-income countries. Extracellular vesicles (EVs) are key mediators of intercellular communication and metabolic reprogramming, but their molecular cargo in EC remains poorly characterized. EVs were isolated from four EC cell lines representing Type I and Type II subtypes (AN3CA, ISHIKAWA, HEC1A, and KLE). Untargeted metabolomics was performed by HILIC-LC-MS/MS, proteomics by data-independent acquisition (DIA) mass spectrometry, and multi-omics integration using MetaboAnalyst and OmicsNet. Metabolomic profiling identified 1463 annotated features and revealed significant differences among EC cell lines (PERMANOVA, *p* = 0.002). Twenty-eight differentially abundant metabolites, including lactic acid, succinic acid, and uric acid, were identified. Proteomic analysis quantified 8513 proteins with subtype-specific expression patterns. Integrated analysis revealed seven significantly enriched pathways, including glycolysis/gluconeogenesis, central carbon metabolism in cancer, and the pentose phosphate pathway. Increased LDHA abundance in metastatic AN3CA-derived EVs was confirmed by Western blot (*p* = 0.047). EC-derived EVs display subtype- and metastatic-status-specific metabolo-proteomic signatures, with glycolysis, TCA cycle remodeling, and central carbon metabolism as convergent pathway signatures of molecular reprogramming. These findings establish a multi-omics framework for characterizing EV cargo in EC and identify candidate enzyme–metabolite nodes for future biomarker validation in patient-derived specimens.

## 1. Introduction

Endometrial cancer (EC) is the most common gynecological malignancy in high-income countries and the sixth most common cancer among women worldwide, with approximately 400,000 new cases diagnosed annually according to GLOBOCAN 2020 estimates [1]. Its incidence has increased by 132% over the last three decades, largely driven by rising obesity rates and population aging [2], making EC an increasingly significant public health concern. Traditionally, EC has been classified into Type I (endometrioid, low-grade, hormone-dependent, favorable prognosis) and Type II (serous, clear cell, carcinosarcoma, high-grade, poorer prognosis) tumors [3]. More recently, the 2023 FIGO staging revision incorporated the TCGA molecular classification (POLE ultramutated, MSI-H/MMRd, NSMP, p53abn), enabling more accurate prognostic stratification compared with the 2009 system [3,4].

Beyond histopathological and molecular classification, increasing evidence indicates that metabolic alterations play a critical role in EC progression and may provide additional biomarkers and therapeutic targets. Metabolic reprogramming is a well-established hallmark of cancer [5]. Tumor cells preferentially rely on aerobic glycolysis (the Warburg effect), promoting survival, proliferation, and treatment resistance [5,6]. In EC, metabolic reprogramming is closely associated with metabolic syndrome, including obesity, hyperglycemia, and dyslipidemia, which represent major risk factors for the disease [7]. Distinct metabolic adaptations have been identified among EC cell subtypes, particularly in cancer stem cells and chemotherapy-resistant cells, highlighting an important role for both glycolysis and oxidative phosphorylation (OXPHOS) [7].

In recent years, extracellular vesicles (EVs) have emerged as key mediators of intercellular communication within the tumor microenvironment and at distant sites [8,9]. EVs are lipid bilayer particles released by all cells, classified based on their size or composition according to the MISEV2023 guidelines [10,11]. Tumor-derived EVs carry a heterogeneous cargo of proteins, nucleic acids, lipids, and metabolites that reflects the molecular state of the parent cell and can modulate signaling pathways, angiogenesis, metastasis, immune evasion, and tumor microenvironment plasticity in recipient cells [8,9]. Beyond serving as passive biomarkers, growing evidence indicates that EVs actively drive metabolic reprogramming. For example, EVs derived from prostate cancer cells have been shown to induce a Warburg-like glycolytic phenotype in recipient cells [12], while tumor-derived EVs can mediate metabolic rewiring in distant organs such as the liver [13]. This effect is mediated by the transfer of metabolic enzymes, including LDH, PKM2, and HK1/2, as well as metabolites and regulatory nucleic acids capable of modulating glycolytic activity in target tissues [9]. Their metabolomic cargo includes TCA cycle intermediates, purines, amino acids, bioactive lipids, and pentose phosphate pathway metabolites, making EV metabolomics a valuable complement to proteomic analyses [3,14].

In EC, the molecular characterization of EVs remains limited, although recent studies by us and others have identified subtype-specific proteomic signatures and obesity-associated differences in EV cargo [15,16]. These findings highlight the complexity of EV-mediated tumor biology and underscore the need for multi-omics approaches to achieve a more comprehensive understanding of their functional role [3]. Because no single omics platform can fully capture the molecular alterations underlying tumor reprogramming, integrating proteomic and metabolomic data has emerged as a powerful strategy to reconstruct enzyme–metabolite networks, identify key molecular hubs and metabolic bottlenecks, and reveal convergent pathways involved in cancer progression [17].

Given the increasing incidence of EC, the emerging role of EVs in metabolic reprogramming, and the availability of advanced multi-omics tools, this study integrates proteomic and metabolomic data from EVs derived from four EC cell lines (AN3CA, ISHIKAWA, HEC1A, and KLE, representative of Type I and Type II endometrial cancer subtypes and of both metastatic and non-metastatic disease) to reconstruct enzyme–metabolite interaction networks and identify convergent pathway signatures underlying the molecular reprogramming of EC-derived EVs.

## 2. Results

### 2.1. Extracellular Vesicle Characterization

**Figure 1:**
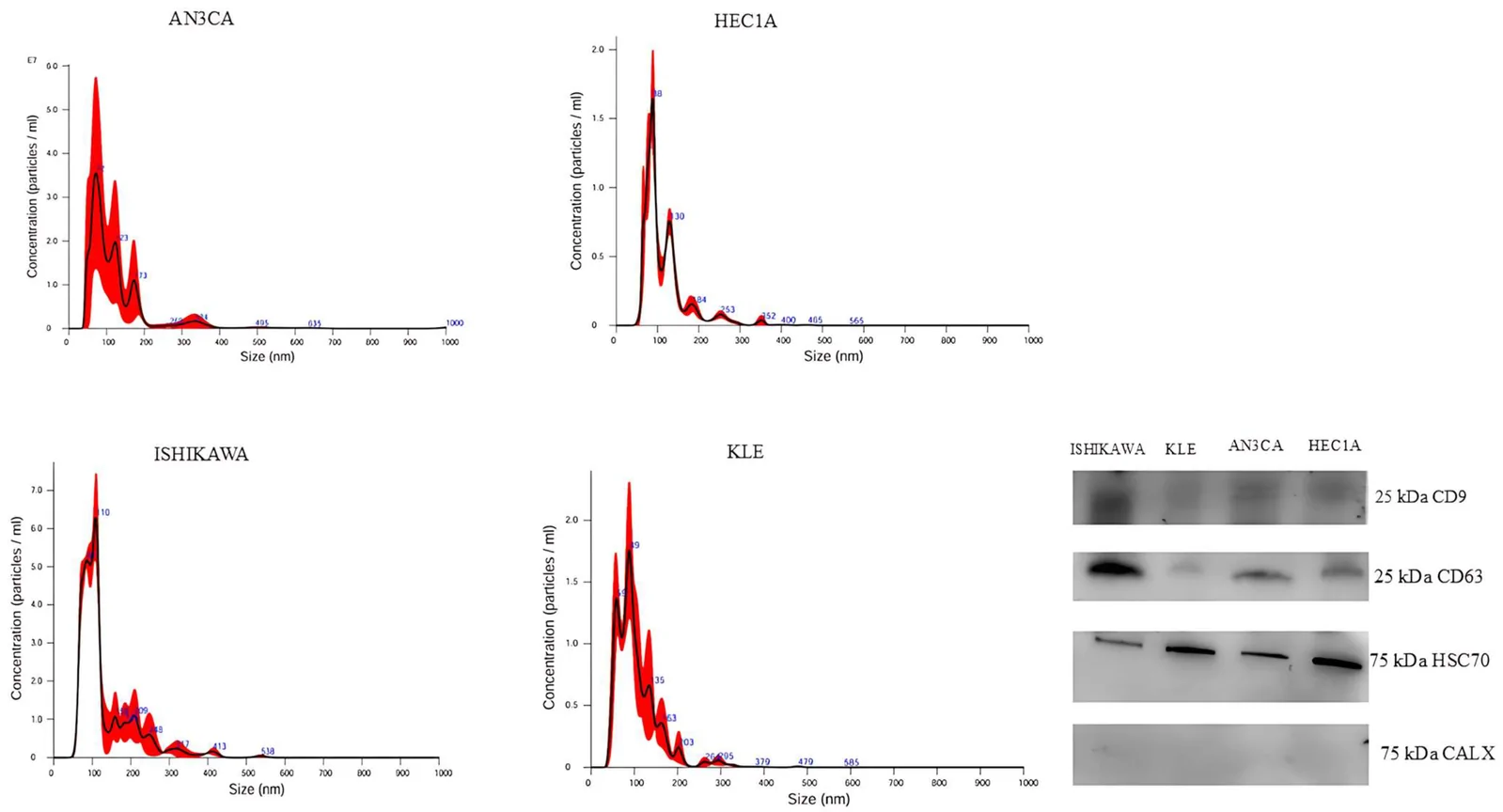
A comprehensive morphological and biochemical characterization of EVs derived from endometrial cancer cell lines. The nanoparticle tracking analysis (NTA) size distribution and concentration profiles of EVs isolated from four EC cell lines: AN3CA, HEC1A, ISHIKAWA, and KLE. For each cell line, the black line represents the mean particle size distribution, with the shaded area representing the standard error of the mean across replicates. The *x*-axis displays particle diameter (nm), and the *y*-axis shows particle concentration (particles/mL). All four cell lines successfully produced EVs with a predominant peak in the 50–150 nm size range, classifying them as small extracellular vesicles (sEVs) in accordance with MISEV guidelines. The right panel shows Western blot analysis supporting robust EV enrichment and purity across all four cell lines (ISHIKAWA, KLE, AN3CA, HEC1A). The expression of canonical EV-positive markers CD9 (~25 kDa) and CD63 (~25 kDa) and the cytosolic EV marker HSC70 (~75 kDa) was confirmed. The absence of the endoplasmic reticulum-negative marker CALX (~75 kDa) demonstrates the effective depletion of intracellular cellular contaminants.

To study EVs from EC, four different EC cell lines, Type I (Ishikawa and AN3CA) and Type II (HEC1A and KLE), were used. These lines represent both primary and metastatic sources. We isolated EVs from the conditioned media and characterized them with biophysical and biochemical methods. Nanoparticle tracking analysis (NTA) showed differences in EV concentration and size distribution among the cell lines (Figure 1). AN3CA-derived EVs had a concentration of 2.69 × 10^9^ particles/mL and a modal size of 71.4 nm. HEC1A-derived EVs had a concentration of 8.18 × 10^8^ particles/mL and a modal size of 87.8 nm. Ishikawa-derived EVs showed a concentration of 3.27 × 10^8^ particles/mL with a modal size of 120.8 nm, while KLE-derived EVs had a concentration of 1.2 × 10^9^ particles/mL and a modal size of 88.3 nm. To confirm the isolation of EVs, and the purity of EVs in accordance with MISEV guidelines, we performed Western blot analysis for common exosomal positive markers like CD63 and CD9, along with the EV-associated cytosolic protein HSC70. Furthermore, we evaluated the expression of the endoplasmic reticulum marker calnexin (CALX) as a negative control. The expression of positive markers and the absence of CALX demonstrated the successful depletion of intracellular organelle contaminants.

Overall, these findings suggest a successful enrichment in exosome-like vesicles from all EC cell lines.

### 2.2. Untargeted Metabolomic Analysis of EVs

**Figure 2:**
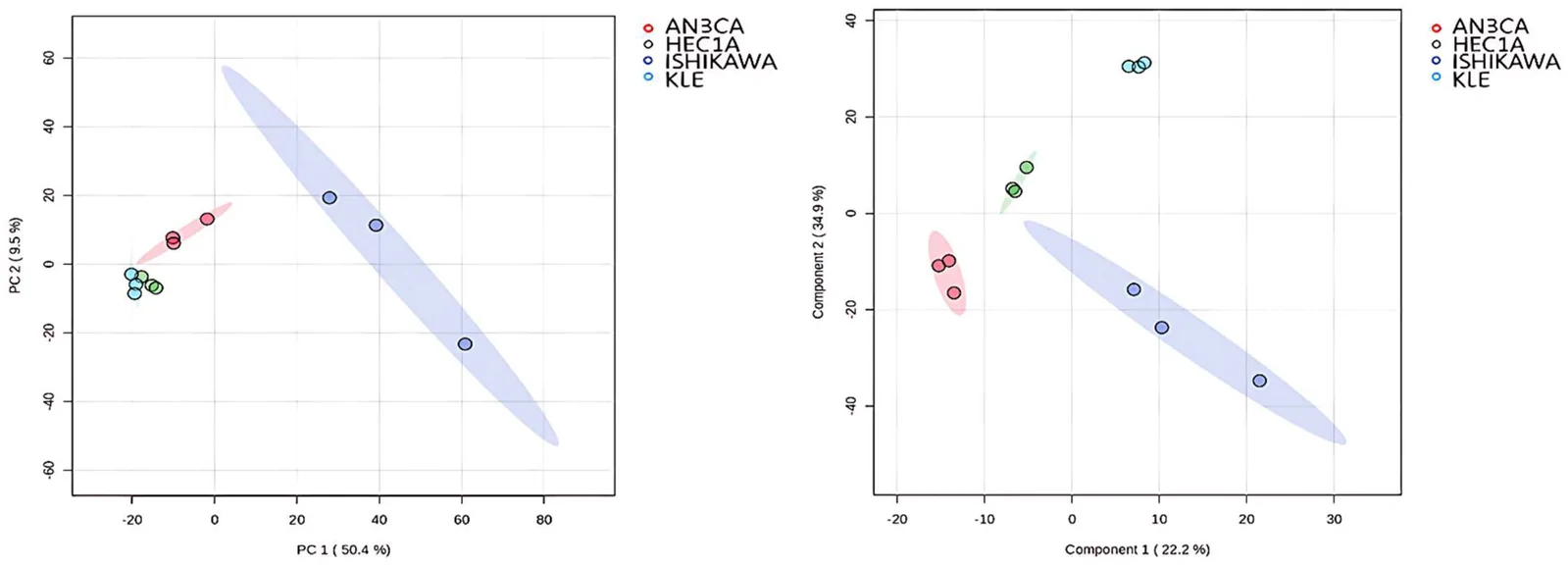
The multivariate analysis of negative metabolites in cell line EVs. Unsupervised Principal Component Analysis (PCA) (**left panel**) displays the spatial separation between EV metabolites derived from AN3CA (red circles), HEC1A (green circles), ISHIKAWA (blue circles), and KLE (azure circles). The PCA highlights that biological variance drives the clustering, with PC1 (50.4%) and PC2 (9.5%) collectively explaining 59.9% of the total variance. The (**right panel**) displays a supervised Partial Least Squares Discriminant Analysis (PLS-DA) score plot, which shows increased discrimination and class clustering among the four groups, with the first two components explaining 57.1% of the total variance. The shaded ellipses represent the 95% confidence intervals for each sample group, underscoring the high reproducibility of the distinct metabolic reprogramming within each EC subtype.

All EV preparations were subjected to LC-MS/MS. Mass spectrometry identified 313,938 features for positive ionization ESI+ with the sample in three biological replicates. Feature intensities were averaged across replicates. Data were filtered by excluding features lacking MS/MS information and/or metabolite annotation and applying a mass accuracy threshold of ±5 ppm. After filtering, 1927 MS/MS-supported metabolite annotations were retained. Multivariate analysis revealed low discrimination among the four EC cell lines (PERMANOVA: F = 0.54538; R^2^ = 0.16979; *p* = 0.10, 999 permutations) (Supplementary Figure S1). Therefore, all subsequent analyses and biological interpretation were focused on the ESI− data. We identified 53,495 features from the negative ionization analysis ESI−. The application of identical positive ionization filters to our dataset resulted in the identification of 1463 MS/MS-annotated metabolites, which were retained for downstream analyses. As for PCA, Figure 2 reveals a clear separation among the four EV cell lines. PC1 (50.4%) and PC2 (9.5%) together explained 59.9% of the total variance. The PERMANOVA test confirmed highly significant differences between cells (F = 12.588; R = 0.82519; *p* = 0.002). PLS-DA (used as an exploratory visualization tool) showed an even clearer distinction between the cell EVs, with the first two components explaining 57.1% of the variance (Component 1: 22.2%; Component 2: 34.9%).

**Figure 3:**
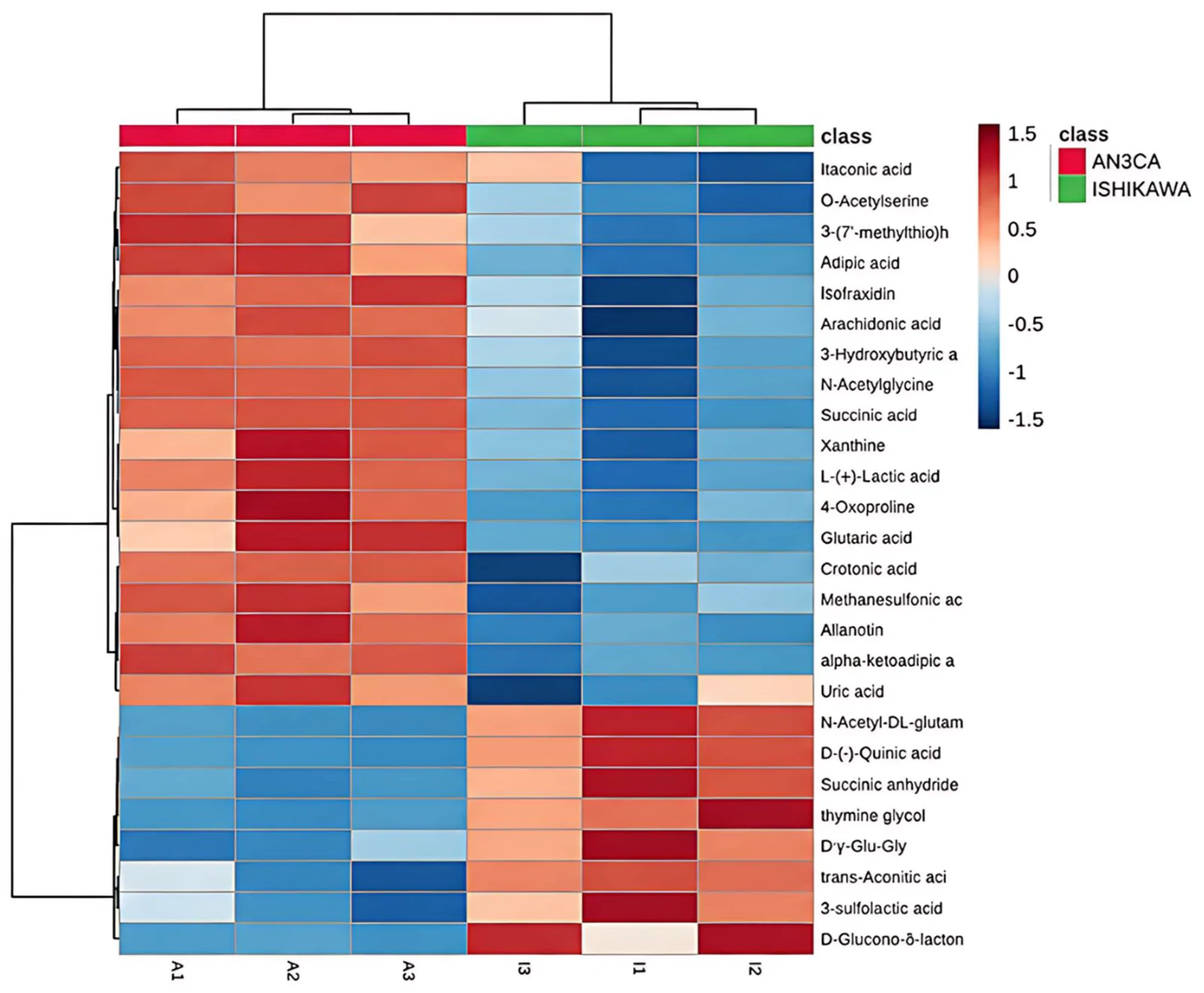
The hierarchical clustering of EV metabolomic profiles. The heatmap illustrates the relative abundance profiles of the selected differentially abundant metabolites identified via the LC–MS/MS metabolomic analysis of the EV preparations. The columns represent individual biological replicates (AN3CA in red and ISHIKAWA in green), and the rows represent the annotated metabolites. Relative metabolite abundance is represented by a robust color gradient (Z-score), ranging from blue (low abundance) to dark red (high abundance). Unsupervised hierarchical clustering (shown as dendrograms on the rows and columns) highlights the perfect grouping of samples based on their cell of origin, visually emphasizing the profound metabolic contrast between the metastatic (AN3CA) and non-metastatic (ISHIKAWA) Type I EV molecular cargo.

Furthermore, we applied a *t*-test (*p* < 0.05) combined with a fold change threshold of ≥1.5 or ≤0.66 to determine significantly abundant metabolites. Following this comparison, we identified 26 (Supplementary File S1) differentially abundant metabolites in the AN3CA/ISHIKAWA EVs represented in the heatmap (Figure 3). Meanwhile, there are two differentially abundant metabolites between KLE/HEC EVs L-(−)malic acid and 12-HETE, which are both more abundant in HEC. Upon applying the stringent Benjamini–Hochberg false discovery rate (BH-FDR) correction (q < 0.05) to the metabolomic dataset, succinic acid remained the only statistically significant altered metabolite. However, to facilitate a comprehensive cross-omics comparison and pathway integration, an exploratory signature based on nominal *p* < 0.05 and fold change >1.5 or ≤0.66 was utilized.

Two main abundance patterns were observed: a cluster of metabolites showing higher abundance in AN3CA in red including uric acid, succinic acid, allantoin, L-(+)-lactic acid, and 3-hydroxybutyric acid and a second cluster showing higher abundance in ISHIKAWA including trans-aconitic acid, 3-sulfolactic acid, D-γ-Glu-Gly, D-glucono-δ-lactone, and succinic anhydride. Sample clustering confirmed a clear separation between AN3CA and ISHIKAWA EVs.

**Figure 4:**
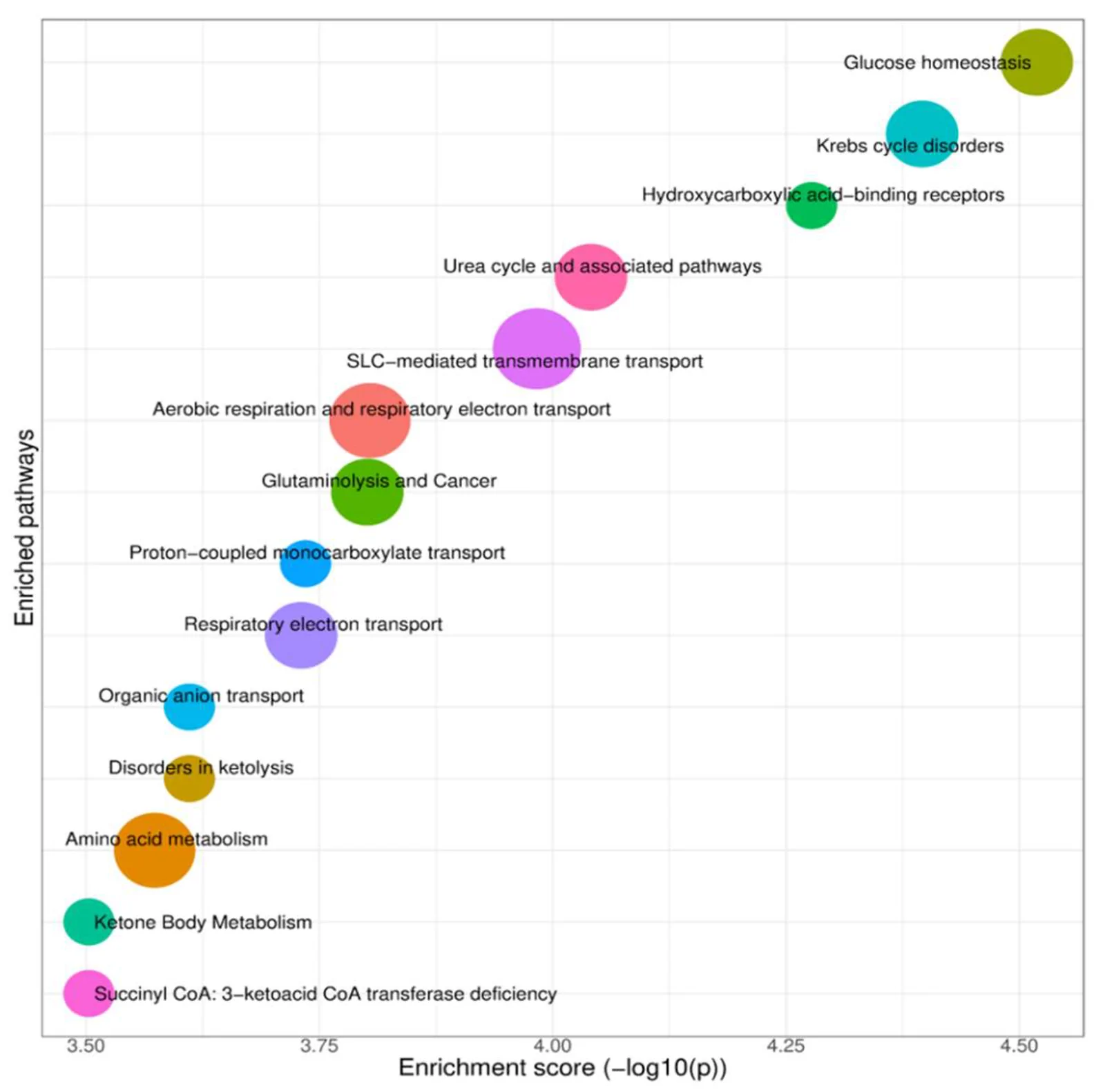
The metabolic enrichment analysis and functional network of differentially abundant EV metabolites. Enriched metabolic pathways, generated from the targeted exploratory metabolic signature, are ranked by their statistical significance on the *y*-axis and represented by their −log10(*p*-value) on the *x*-axis. The size of the bubbles corresponds to the enrichment ratio (impact score) for each given pathway. This functional landscape highlights a pronounced rewiring of energy- and transport-related metabolic pathways across the EV cell lines, including prominent enrichment in glucose homeostasis, Krebs cycle disorders, and amino acid metabolism. This comprehensive view of the data further supports the findings from the multivariate and heatmap analyses, demonstrating that the robust separation between the cell lines is driven by a consistent and biologically meaningful metabolic reprogramming.

We continued with the enrichment analyses using the Metaboanalyst 6.0 Enrichment Pathway module on the 28 differentially abundant metabolites (Figure 4). The pathway enrichment analysis of metabolites derived from the extracellular vesicles (EVs) of endometrial cancer cell lines revealed significant alterations mainly associated with energy metabolism and mitochondrial function. The most enriched pathways included glucose homeostasis, Krebs cycle disorders, and respiratory electron transport, suggesting metabolic rewiring and altered bioenergetic activity in EV-associated metabolites. In addition, pathways related to amino acid metabolism, ketone body metabolism, and transmembrane transport were significantly represented, indicating a broad modulation of metabolic processes in EC-derived EVs.

### 2.3. EV Proteomic Study

**Figure 5:**
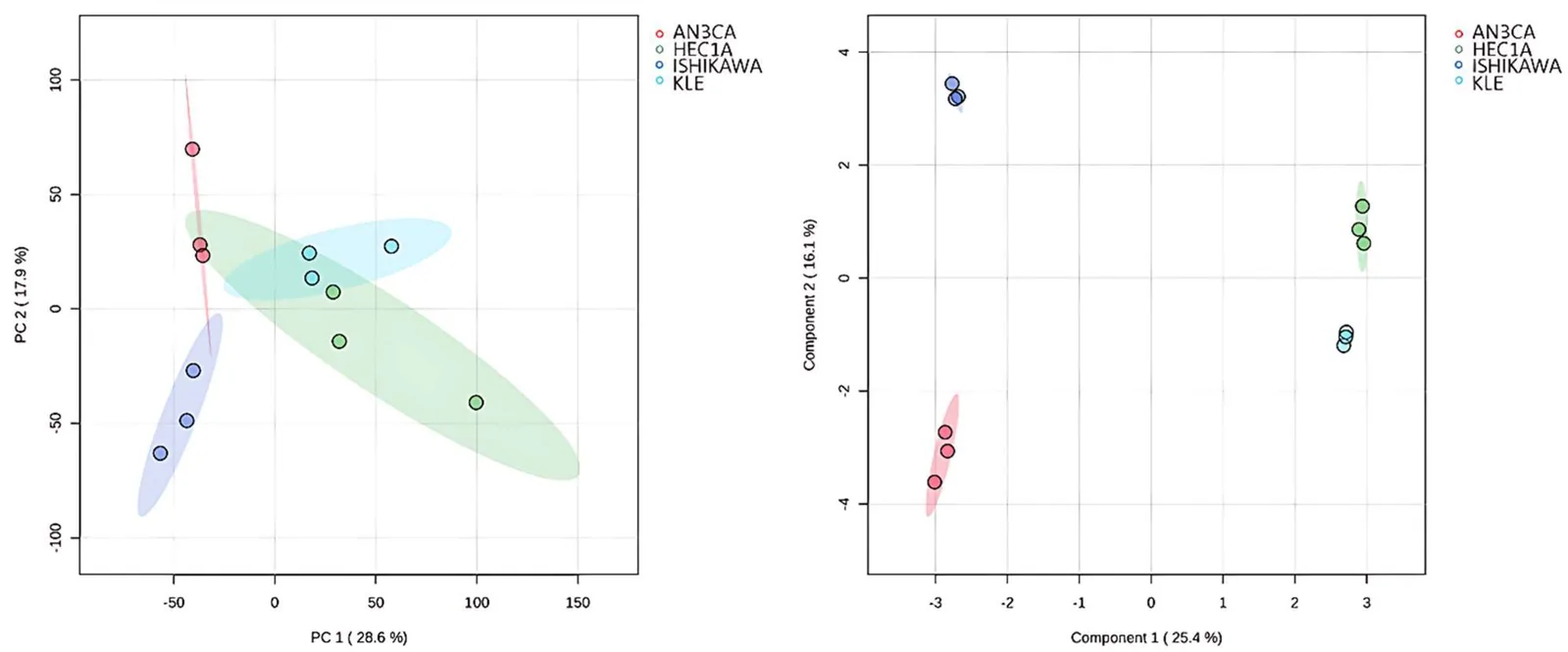
The multivariate statistical analysis of the deep EV proteomic profile. Unsupervised PCA (**left panel**) demonstrates a clear separation between the comprehensive EV proteomes of AN3CA (red circles), HEC1A (green circles), ISHIKAWA (blue circles), and KLE (azure circles) based on DIA mapping 8513 strictly filtered proteins. PC1 (28.6%) and PC2 (17.9%) explain a combined 46.5% of the total variance. The PLS-DA score plot (**right panel**) illustrates the enhanced, supervised separation of the groups, with the first two components explaining 41.5% of the variance. The colored ellipses represent the 95% confidence area for each respective group. The high variance explained by the primary components confirms that intrinsic biological differences, rather than technical variability, dictate the EV proteomic clustering.

The protein composition of the EVs from EC cells (the same used in the metabolomic study) was analyzed. For DIA file processing, we used Spectronaut 20 and identified 9962 protein groups across three biological replicates per cell line, with q < 0.01 (1% FDR). To increase specificity for EV-associated proteins, we applied stringent filtering by removing proteins reported as common EV contaminants according to MISEV2023, as well as protein groups lacking gene annotation, putative/probable/similar entries, cDNA-derived entries, keratins, and proteins with missing values. This yielded a final dataset of 8509 proteins, which is provided as Supplementary File S2. Proteomic analysis revealed differences between the four EC cell line EVs. PCA shows good separation (Figure 5) between the four EV cell lines, with PC1 (28.6%) and PC2 (17.9%) together explaining 46.5% of the variance. The PERMANOVA confirmed significant overall differences between the groups (F = 12.448, R^2^ = 0.82, *p* = 0.001, 999 permutations). PLS-DA was used only as an exploratory visualization tool. No inferential conclusions were drawn from the PLS-DA model due to the limited sample size; statistical inference relied on PCA/PERMANOVA and univariate tests.

**Figure 6:**
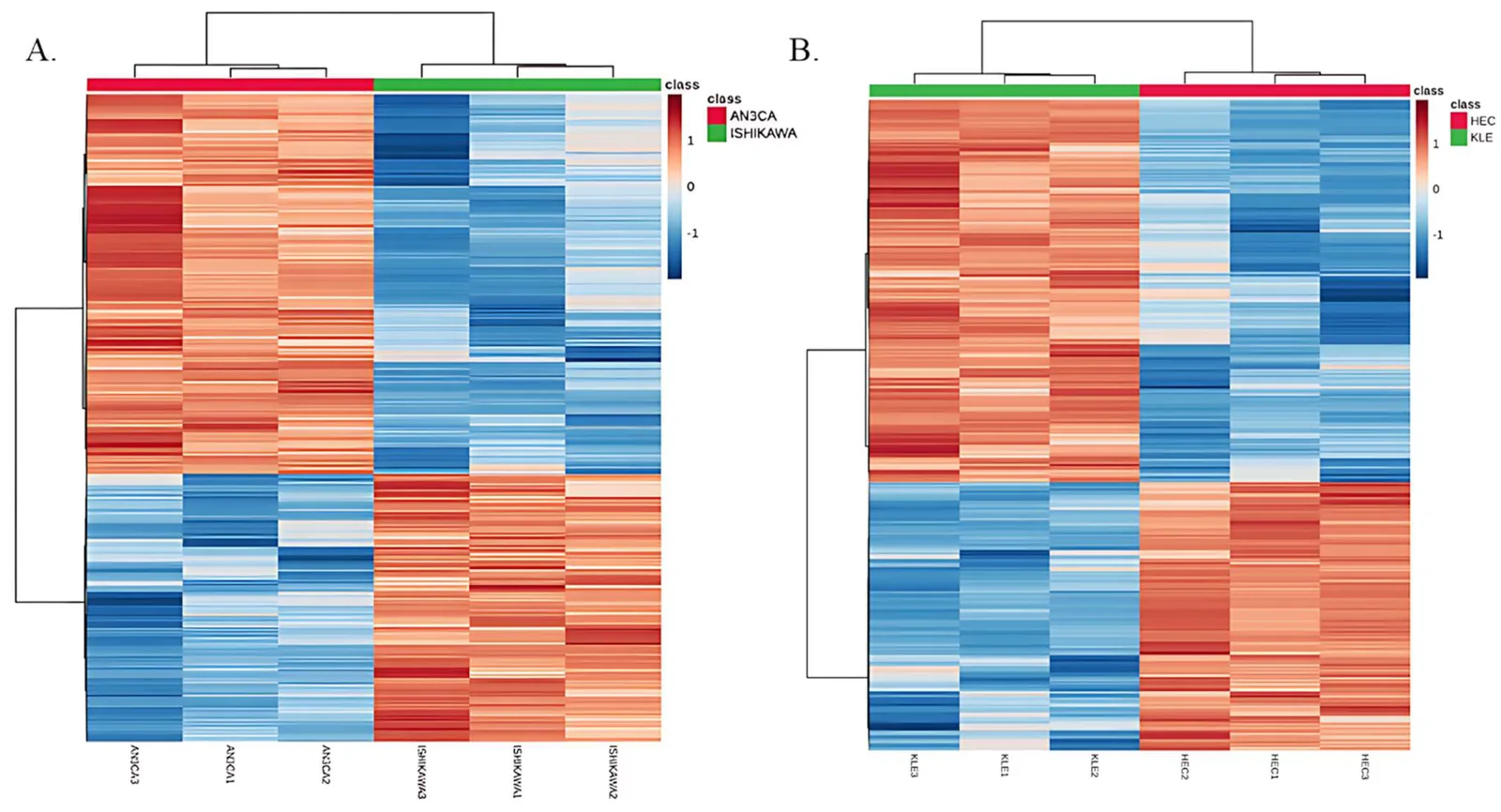
The hierarchical clustering heatmap of differentially abundant EV proteins. Unsupervised hierarchical clustering was performed to visualize proteomic variations in (**A**) Type I (AN3CA vs. ISHIKAWA) and (**B**) Type II (KLE vs. HEC1A) EC EV cell line comparisons. Given the exploratory nature of this study and the aim of pathway-based multi-omics integration, differentially abundant proteins were selected based on a fold change >1.5 or <0.66 and a nominal *t*-test *p*-value < 0.05. This threshold identified an exploratory signature of 975 differentially abundant proteins for the AN3CA/ISHIKAWA comparison and 533 proteins for the KLE/HEC1A comparison. Individual biological replicates are represented as columns, and significantly altered proteins are represented as rows. Protein abundance is depicted by a standardized color gradient ranging from blue (low abundance) to brown/red (high abundance). Dendrograms on both axes illustrate the unsupervised hierarchical clustering of samples and proteins, demonstrating a clear separation between cell lines in both comparisons.

Overall, these results support the existence of a distinct proteomic profile between the EVs. All statistical tests were performed at the level of biological replicates (n = 3 per cell line), without the prior aggregation of replicates. Applying a fold change ≥1.5 or ≤0.66 and a *t*-test *p*-value < 0.05 to the 8509 proteins resulted in 975 proteins with differential abundance in AN3CA/ISHIKAWA and 533 proteins with differential abundance in KLE/HEC. The heatmap plot (Figure 6) shows separation between EVs. Using BH-FDR correction (q < 0.05) (Supplementary File S3), 74 proteins remained significant in KLE/HEC1A, while 46 proteins remained significant in AN3CA/ISHIKAWA (Supplementary File S4).

**Figure 7:**
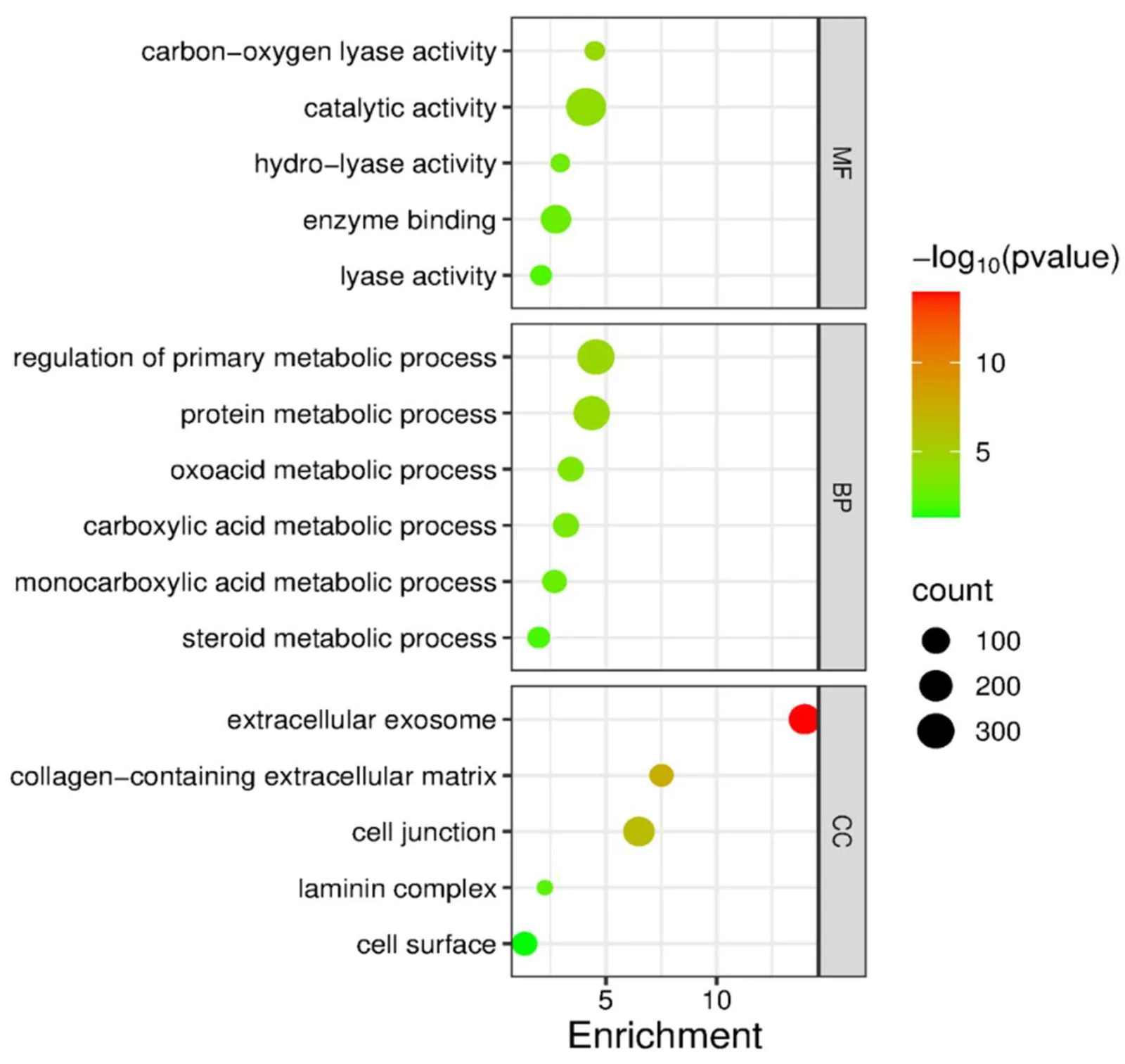
A comprehensive functional Gene Ontology (GO) enrichment analysis of EV proteomes. The g:Profiler results displaying the primary GO terms across biological process (BP), Cellular Component (CC), and molecular function (MF) derived from the differentially abundant EV proteins. Enriched terms are sorted by their robust statistical significance (*x*-axis: −log10(*p*-value)), with the color gradient ranging from green (moderately significant) to red (highly significant). Dot size directly corresponds to the number of individual proteins annotated to each specific term. The significant enrichment in the “extracellular exosome” CC term validates the vesicular nature of the analyzed material, while terms such as “catalytic activity” and “oxoacid metabolic process” reveal a vast cargo of metabolically active EV-associated enzymes.

Given the small sample size and the aim of hypothesis generation and pathway-based multi-omics integration, we additionally considered a broader set of proteins meeting the criteria of a fold change ≥1.5 or ≤0.66 and nominal *p* < 0.05 as an exploratory signature. This exploratory set was used to identify enzyme-annotated proteins for integration with metabolomics. To characterize the biological significance of the 1290 candidate proteins, a functional enrichment analysis was performed using g:Profiler (Figure 7).

The Gene Ontology enrichment analysis of proteins identified in EVs derived from EC cell lines revealed a significant enrichment in terms associated with extracellular exosomes, cell junctions, and extracellular matrix components, including collagen-containing extracellular matrix and laminin complex. Biological processes were mainly related to metabolic and protein metabolic processes, particularly carboxylic acid and oxoacid metabolism, suggesting the active involvement of EV proteins in metabolic regulation. In addition, molecular function analysis highlighted catalytic and enzyme-binding activities, supporting the presence of metabolically active and regulatory proteins within tumor-derived EVs.

### 2.4. Multi-Omics Data Integration

**Figure 8:**
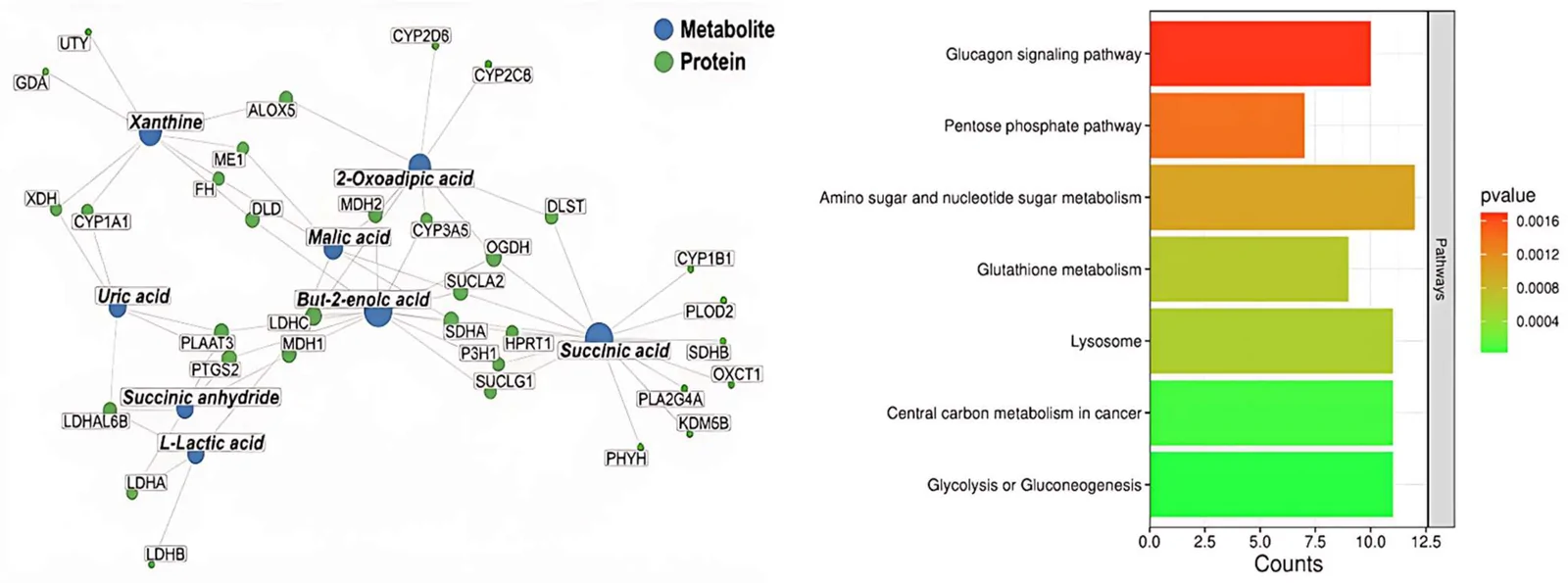
The multi-omics integration of EV proteomic and metabolomic data. (**Left Panel**) A knowledge-based molecular interaction network created using OmicsNet, directly mapping the intricate crosstalk between differentially abundant EV proteins and metabolites. Nodes represent specific proteins (in green) and identified metabolites (in blue). Edges denote validated biochemical interactions sourced from primary biological databases, clustering around key central hubs such as succinic acid, malic acid, and L-lactic acid. (**Right Panel**) A joint pathway analysis performed in MetaboAnalyst, combining both omics datasets to identify the most significantly enriched shared metabolic pathways (BH-FDR q < 0.05). Bars display the number of matched multi-omics features for each pathway. Color indicates statistical significance, ranging from green to red with greater significance, underscoring critical EV-mediated alterations in glycolysis/gluconeogenesis, central carbon metabolism, and glucagon signaling pathways.

Using the PANTHER classification system, 344 enzymes were identified among the 1290 significantly altered proteins. These enzymes were then integrated with the 28 differentially abundant metabolites identified across both comparisons (26 from AN3CA/ISHIKAWA and 2 from KLE/HEC1A). To perform the integrated metabolomic and proteomic analysis, the OmicsNet and MetaboAnalyst platforms were used (Figure 8). Pathway enrichment analysis was performed using MetaboAnalyst. This analysis showed that seven pathways were significantly enriched. These pathways were glycolysis/gluconeogenesis, central carbon metabolism in cancer, the glucagon signaling pathway, amino sugar and nucleotide sugar metabolism, glutathione metabolism, the lysosome and the pentose phosphate pathway. The results were consistent with the metabolomic–proteomic interaction network that we constructed using OmicsNet.

The network is made up of the top eight metabolites and 33 enzymes to simplify the analysis. Certain metabolites, such as succinic acid and malic acid, were key hubs that interacted with important TCA cycle enzymes like SDHA, SDHB, MDH1, MDH2, OGDH, DLST and FH. This highlighted the central carbon metabolism in cancer pathway, underscoring the fundamental role of carbon metabolism in cancer. Furthermore, a distinct cluster was identified, centered on L-lactic acid and different lactate dehydrogenase isoforms such as LDHA, LDHB, LDHC and LDHAL6B. This was consistent with the glycolysis/gluconeogenesis pathway enrichment, consistent with the activation of the Warburg effect. We also found a purine catabolism cluster that involved xanthine, uric acid, xanthine dehydrogenase and hypoxanthine–guanine phosphoribosyltransferase 1 which further verified the identification of the pentose phosphate pathway. In addition, we found that ALOX5 prostaglandin–endoperoxide synthase 2 and phospholipase A2, group IVA were involved in stress and inflammatory signaling activation. Our analyses confirmed that our identified multi-omics signature was reliable. Taken together, these data suggest a metabolic rewiring involving the central carbon metabolism in cancer and glycolysis/gluconeogenesis pathways.

### 2.5. Western Blotting

**Figure 9:**
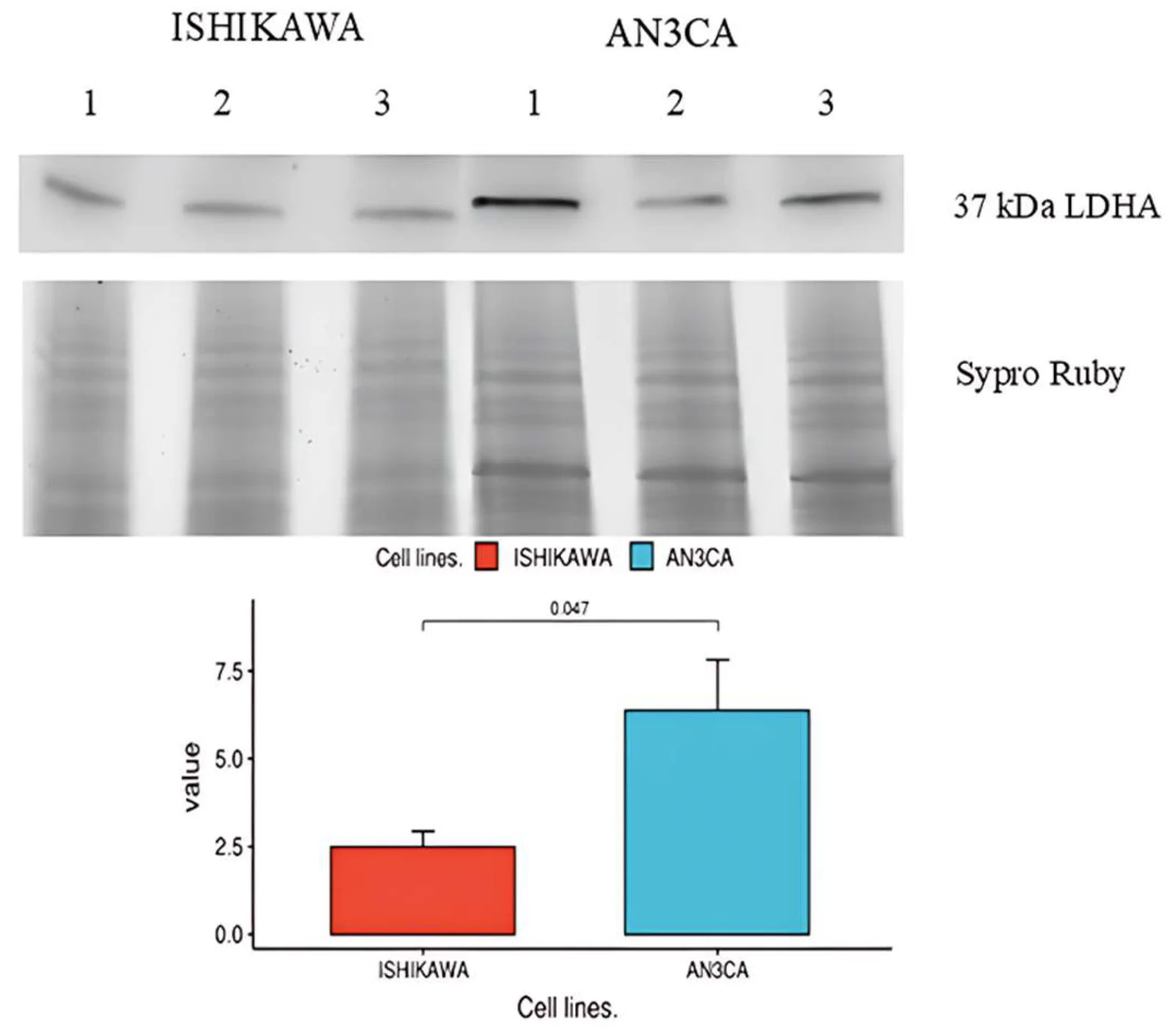
Western blot analysis validating the differential abundance of LDHA between Ishikawa and AN3CA EVs. Representative LDHA immunoblots are shown in the (**upper panel**). LDHA was selected specifically because it met strict differential abundance criteria exclusively in the Type I omics comparison. LDHA band intensities were quantified by densitometry and precisely normalized to the total protein loading, which was determined by the SYPRO Ruby staining of a duplicate gel run in parallel and loaded with the exact same samples (**middle panel**). Densitometric analysis (**bottom panel**) confirmed a significantly higher abundance of LDHA in AN3CA EVs compared with Ishikawa EVs (unpaired *t*-test, *p* = 0.047), directly corroborating the multi-omics integration network. Data are presented as the mean ± standard deviation (SD) derived from three independent biological replicates.

To further support the proteomic data, LDHA protein expression was validated by Western blot analysis (Figure 9) (Supplementary Figure S2). LDHA was selected as the primary validation target given its central role in both the glycolysis/gluconeogenesis pathway and the lactate metabolism network identified by multi-omics integration and given its direct interaction with L-lactic acid in the OmicsNet network. Furthermore, this validation was specifically focused on the AN3CA vs. ISHIKAWA comparison because LDHA successfully met our predefined statistical threshold for differential abundance (*t*-test *p* < 0.05 and FC ≥ 1.5) exclusively in this Type I cell line pairing, whereas it did not pass the significance filter in the Type II (KLE vs. HEC1A) proteomic dataset.

Statistical analysis using an unpaired *t*-test revealed a significant increase in LDHA abundance (*p* = 0.047) in AN3CA EVs compared to ISHIKAWA EVs, successfully corroborating the proteomic findings.

## 3. Discussion

This study provides an integrated proteomic and metabolomic characterization of extracellular vesicles (EVs) derived from four endometrial cancer (EC) cell lines representing Type I and Type II subtypes. By combining untargeted metabolomics, DIA, and multi-omics network integration, we identified EV-associated molecular signatures indicative of metabolic reprogramming involving glycolysis, the TCA cycle, purine catabolism, and oxidative stress.

Successful EV isolation and characterization from all four EC cell lines were confirmed by nanoparticle tracking analysis (NTA) and Western blotting. NTA demonstrated that all isolated vesicles had diameters below 200 nm, classifying them as small extracellular vesicles (sEVs). However, despite belonging to the same size category, substantial differences in particle size distribution were observed among the cell lines. In particular, the modal size varied considerably, ranging from 71.4 nm in AN3CA to 120.8 nm in ISHIKAWA (with ISHIKAWA-derived vesicles being almost twice the size of those released by AN3CA). Furthermore, the inspection of the NTA profiles together with the variable expression of EV protein markers across the four cell lines suggests the presence of heterogeneous EV populations. This heterogeneity may reflect differences in EV biogenesis and/or cellular origin. Supporting this hypothesis, pathway enrichment analysis revealed that, among the seven significantly enriched pathways, the lysosome was the only pathway not directly related to metabolic processes. Rather than representing a metabolic alteration, its enrichment may reflect the endosomal biogenesis of EVs through the multi-vesicular body (MVB) pathway and the contribution of lysosomal exocytosis to EV secretion. In fact, lysosomal exocytosis has been identified as one of the basic processes whereby lysosomes fuse with the plasma membrane, followed by the discharge of their contents into the extracellular environment with an emerging role in the biogenesis and secretion of EVs [18].

Of all the four EC cell lines, the maximum amount of EVs was secreted by AN3CA, while the minimum amount was secreted by ISHIKAWA. The above results correlate with the widely known relationship between EV secretion and the aggressiveness of tumors. Highly aggressive cancer cells undergo profound metabolic reprogramming characterized by enhanced aerobic glycolysis (the Warburg effect), which generates the energy and biosynthetic precursors required to sustain rapid proliferation. This elevated metabolic activity is also accompanied by increased EV biogenesis and secretion, enabling tumor cells to remodel the tumor microenvironment, facilitate intercellular communication, and promote cancer progression. Therefore, the higher EV yield observed in AN3CA cells, compared with the lower secretion detected in ISHIKAWA cells, likely reflects the greater metabolic activity and more aggressive phenotype of AN3CA, in agreement with previous reports linking enhanced EV release to metabolically active tumors [19].

The presence of canonical EV markers in all samples indicates that the samples are reliable and comparable for further multi-omics analysis.

Metabolomic profiling (PCA, PLS-DA, and PERMANOVA) further revealed distinct EV metabolic signatures across EC cell lines. AN3CA EVs were enriched in uric acid, succinic acid, allantoin, L-(+)-lactic acid, and 3-hydroxybutyric acid, supporting the coexistence of glycolytic and mitochondrial metabolism characteristic of metabolically plastic, aggressive cancer cells [20]. Uric acid and allantoin, both products of purine catabolism, further indicate active nucleotide turnover, likely linked to the high proliferative demand of metastatic cells and to oxidative stress responses [21]. Conversely, ISHIKAWA EVs were enriched in trans-aconitic acid, 3-sulfolactic acid, D-γ-Glu-Gly, D-glucono-δ-lactone, and succinic anhydride. The enrichment in trans-aconitic acid, a TCA cycle-related metabolite, and D-γ-Glu-Gly, associated with glutathione metabolism, suggests a more oxidative mitochondrial phenotype and distinct antioxidant response in non-metastatic Type I cells [22]. The pathway enrichment analysis of the 28 differentially abundant metabolites revealed an enrichment in glucose homeostasis, Krebs cycle disorders, aerobic respiration, respiratory electron transport, ketone body metabolism, amino acid metabolism, and transmembrane transport. Together, these findings support a hybrid metabolic state in EC-derived EVs, reflecting the metabolic plasticity of aggressive cancer cells that can switch between glycolysis and oxidative phosphorylation [23].

While robust metabolo-proteomic differences were clearly observed between the Type I cell lines (AN3CA vs. ISHIKAWA), the multi-omics signatures distinguishing the Type II cell lines (KLE vs. HEC1A) showed a more limited degree of divergence. This relative homogeneity in Type II EVs may reflect a combination of biological and technical factors. From a biological standpoint, Type II endometrial cancers are generally high-grade, poorly differentiated, and clinically aggressive, and it is plausible that KLE and HEC1A share a more convergent baseline state of central energy metabolism, resulting in a less heterogeneous EV cargo than that observed in the Type I models. From a technical standpoint, our metabolomic profiling was restricted to negative ionization mode (ESI−), which is well suited for detecting polar metabolites and organic acids involved in glycolysis and the TCA cycle but may be less sensitive to changes in lipid metabolism, amino acids, and polyamines. Given that lipid metabolic reprogramming is often a hallmark of aggressive Type II carcinomas, the limited divergence observed in the Type II EV dataset may partly reflect analytical coverage rather than complete biological similarity. Future studies using dual-polarity metabolomics and targeted lipidomics will be important to further resolve the molecular features of Type II EC EVs.

DIA proteomics identified 8513 proteins across the four EC cell lines after strict filtering. This dataset is one of the largest EV proteomic collections reported for EC so far. PCA and PLS-DA showed clear separation among the four cell lines. This indicates that specific molecular features of EC subtypes are reflected in the EV proteome. Additionally, the high amount of variance explained by the first two principal components suggests that biological differences mainly drove the observed clustering, not technical variability. The Western blot validation of LDHA in AN3CA vs. ISHIKAWA confirmed the biological relevance of the proteomic findings. LDHA is a key regulator of glycolytic metabolism and is frequently overexpressed in a wide range of cancers [24]. In EC, increased LDHA expression has been associated with tumor progression, invasiveness, treatment resistance, and poor prognosis, underscoring its central role in metabolic reprogramming. [25,26]. The consistent upregulation of LDHA observed by both DIA proteomics and Western blot supports enhanced glycolytic reprogramming in metastatic Type I EC-derived EVs and is consistent with previous reports in aggressive EC [27].

The results from the Gene Ontology term enrichment analysis showed that terms associated with extracellular exosomes, cell junctions, and extracellular matrix molecules, such as collagen-rich extracellular matrix and laminin complex, were significantly enriched. These results agree well with the known ability of tumor EVs to remodel the extracellular matrix and create the pre-metastatic niche [28]. The observed enrichment for biological processes associated with carboxylic acid and oxoacid metabolism, as well as for molecular functions like catalytic activity and enzyme binding, clearly indicates the presence of active metabolic enzymes in EVs derived from ECs [29]. While the previous literature suggests that tumor EVs can transfer active enzymes capable of modulating recipient cell bioenergetics [30], our present findings remain correlative. In the absence of direct functional assays, we cannot conclude that these EV-associated enzymes actively alter recipient cell metabolism. Nevertheless, their abundance suggests a potential mechanism for EV-mediated intercellular crosstalk that warrants further investigation.

The integration of the two omics datasets using OmicsNet and MetaboAnalyst indicated seven significantly enriched pathways, including: glycolysis/gluconeogenesis, central carbon metabolism in cancer, the pentose phosphate pathway, glutathione metabolism, glucagon signaling, amino sugar and nucleotide sugar metabolism, and the lysosome. Among the enriched pathways, the lysosome may reflect EV biogenesis through the endosomal/multi-vesicular body (MVB) pathway rather than a tumor metabolic signature, consistent with the established role of lysosomal exocytosis in EV secretion as previously discussed [18].

The convergence of both proteomics and metabolomics indicates that there is coordinated metabolic regulation in EC-derived EVs, involving energy metabolism, redox homeostasis, and biosynthesis [31]. Further network analysis showed connections of the TCA cycle and glycolysis pathways around succinic acid, malic acid, lactate, and their respective enzymes (such as SDH, MDH, OGDH, FH, and different forms of LDH), indicative of the metabolic flexibility of aggressive EC cells [32]. Furthermore, enrichment in purine metabolism (e.g., xanthine, uric acid, xanthine dehydrogenase, and hypoxanthine phosphoribosyltransferase 1) and inflammatory lipid mediators (arachidonic acid, 12-HETE, ALOX5, PTGS2/COX-2, and PLA2G4A) suggests that EC-derived EVs carry the enzymatic and metabolic machinery that could potentially participate in microenvironmental signaling [33,34]. The metabolomic and proteomic patterns found in this research are congruent with earlier evidence of metabolic reprogramming mediated by EVs in gynecological and other solid cancers. The presence of enzymes involved in glycolysis and lactate metabolism in tumor-derived EVs has already been shown for ovarian [35], breast [36], and prostate [37] cancers, pointing to the conservation of glycolysis in EVs in tumor metabolism. In EC, direct comparisons remain limited due to the limited number of published EV metabolomic studies. However, our proteomic findings agree with previous studies, including those from our group, reporting subtype-specific EV proteomic signatures associated with metastatic status and histological grade [38]. By integrating metabolomic and proteomic data, our study further demonstrates that coordinated alterations in glycolysis, central carbon metabolism, redox homeostasis, and nucleotide metabolism converge on common biological pathways, providing a more comprehensive view of the metabolic landscape of EC-derived EVs than either omics approach alone [39].

Despite the biological relevance of these findings, several limitations should be considered. First, this study was performed using established EC cell lines under controlled in vitro conditions, which cannot fully recapitulate the complexity of the in vivo tumor microenvironment such as interactions with stromal and immune cells, hypoxia and nutrient availability. Second, another limitation of this study is the absence of direct functional validation assays. Although our multi-omics approach identified a rich cargo of metabolic enzymes (e.g., LDHA) and metabolites, these findings remain correlative. Specifically, we did not assess EV uptake kinetics, perform recipient cell co-culture experiments, or measure enzymatic activity. Consequently, the hypothesis that EC EVs actively reprogram recipient cell bioenergetics remains to be formally demonstrated. To bridge this gap, future studies will focus on targeted functional assays to determine the precise bioenergetic impact of EV uptake on recipient cells within the endometrial tumor microenvironment. These studies will include fluorescent EV tracking to confirm uptake kinetics in relevant recipient cells, such as endometrial stromal cells or less aggressive EC phenotypes, as well as co-culture systems combined with live cell metabolic flux analysis to quantify changes in glycolysis and oxidative phosphorylation induced by EV internalization. In addition, substrate turnover assays will be required to establish whether EV-associated enzymes, such as LDHA, retain catalytic activity after transfer and can directly contribute to local metabolic reprogramming. Until such functional validation is performed, our multi-omics network should be interpreted as a robust, hypothesis-generating framework describing the metabolic landscape of EC-derived EVs.

Additionally, while our extracellular vesicles were successfully characterized for size, concentration, and protein marker expression in accordance with MISEV guidelines (confirming the enrichment in CD9, CD63, and HSC70 and the absence of CANX), the lack of morphological validation via electron microscopy (e.g., TEM) represents a technical limitation of the current characterization. Finally, the metabolomic analysis was performed exclusively in negative ionization mode (ESI−), as ESI+ data did not provide sufficient discrimination among the cell lines. Although ESI− is well suited for detecting polar metabolites, organic acids, and nucleotides, this approach may have limited the detection of metabolites that ionize preferentially in positive mode, including several amino acids, lipids, and amines. Although run order signal correction was performed using cell-line-specific pooled QCs to monitor intra-group analytical stability, the absence of a single global QC pool containing aliquots from all cell lines, together with the lack of spiked internal standards added before extraction, limited our ability to fully account for potential inter-group analytical effects. Therefore, the comparisons of relative metabolite abundance across different cell line matrices should be interpreted with caution, as differences in matrix composition may contribute to variable ionization efficiency and matrix effects.

Furthermore, although cold organic solvent precipitation substantially reduced the protein content of the metabolomic extracts, polymer-based reagents used for EV isolation, such as PEG, may remain soluble under the extraction conditions and therefore be retained in the metabolomic extracts. Residual polymeric components may contribute to matrix effects and electrospray ionization (ESI) suppression during MS acquisition. This potential analytical interference represents a limitation to consider when profiling metabolites from EV preparations obtained using polymer-based precipitation methods.

## 4. Methods and Materials

### 4.1. Cell Culture

Four human EC cell lines were used in this study, representing both Type I and Type II EC subtypes with distinct metastatic profiles: AN3CA (metastatic Type I), ISHIKAWA (non-metastatic Type I), HEC1A (non-metastatic Type II), and KLE (metastatic Type II). AN3CA and HEC1A were obtained from the American Type Culture Collection (ATCC, Manassas, VA, USA; Cat. No. HTB-111 and HTB-112, respectively); KLE was obtained from the Istituto Zooprofilattico Sperimentale della Lombardia e Emilia Romagna (Cat. No. BSTCL230); and ISHIKAWA was purchased from CliniSciences (Nanterre, France; Cat. No. ABC-TC1320). Each cell line was maintained under specific culture conditions optimized for its growth requirements. AN3CA cells were cultured in Eagle’s Minimum Essential Medium (EMEM); HEC1A in McCoy’s 5a Medium Modified; KLE in a 1:1 mixture of Dulbecco’s Modified Eagle’s Medium (DMEM; 4.0 mM/L L-glutamine, 1.5 g/L sodium bicarbonate, 4.5 g/L glucose, 1.0 mM sodium pyruvate) and Ham’s F12 medium (1.0 mM/L L-glutamine, 1.17 g/L sodium bicarbonate), reflecting the highly glycolytic and metabolically active phenotype of this cell line; and ISHIKAWA in Minimum Essential Medium (MEM) supplemented with 2 mM glutamine and 1% non-essential amino acids (NEAAs). All media were supplemented with 10% fetal bovine serum (FBS), except ISHIKAWA, which was maintained in 5% FBS, and with penicillin and streptomycin (100 IU/mL each). All cell lines were incubated at 37 °C in a humidified atmosphere containing 5% CO_2_. Mycoplasma and bacterial contamination were excluded by both manufacturer certification and weekly in-house PCR testing targeting the conserved regions of the 16S rRNA gene.

### 4.2. EV Isolation

Prior to EV collection, each cell line was passaged and seeded at line-specific densities, calculated from previously established growth rate ratios, so that all cultures reached approximately 90% confluence at the time of harvest; this approach was adopted to minimize confluence-dependent variability in EV secretion between cell types. Cell viability was routinely monitored by visual inspection and by trypan blue exclusion assay, and no reduction in viability was observed for any cell line, which consistently exceeded 90–95% at the time of conditioned medium collection.

Cells were cultured for 48 h in medium supplemented with exosome-depleted fetal bovine serum (FBS) (Gibco, Thermo Fisher Scientific, Waltham, MA, USA; Cat. No. A27208-01). Conditioned medium (CM) was collected and centrifuged at 500× *g* for 5 min at 4 °C to remove cells and cellular debris. The supernatant was subsequently centrifuged at 2000× *g* for 20 min at 4 °C to remove cellular debris and apoptotic bodies. The resulting supernatant was filtered through a 0.22 μm membrane filter to eliminate large vesicles and aggregates. Subsequently, the filtrate was concentrated to a final volume of 1 mL using a 100 kDa molecular weight cut-off (MWCO) ultrafiltration device. EVs were then isolated using the Total Exosome Isolation Reagent from Cell Culture Media (Invitrogen, Thermo Fisher Scientific, Waltham, MA, USA; Cat. No. 4478359) according to the manufacturer’s instructions. Briefly, 500 μL of isolation reagent was added to 1 mL of concentrated conditioned medium and incubated overnight at 2–8 °C. Following incubation, samples were centrifuged at 10,000× *g* for 1 h at 4 °C to pellet EVs. The pellet was resuspended in phosphate-buffered saline (PBS) and further purified by a second ultrafiltration step using a 100 kDa MWCO device to remove residual precipitation reagent and low-molecular-weight contaminants. Since polymer-based precipitation can co-isolate non-vesicular components, downstream analyses were performed on an EV-enriched fraction. The purified EV preparations were immediately processed for downstream proteomic and metabolomic analyses.

### 4.3. Nanoparticle Tracking Analysis

The concentration and size distribution of purified EVs derived from Ishikawa, AN3CA, HEC1A, and KLE cell lines were determined by nanoparticle tracking analysis (NTA) using a NanoSight LM10 instrument (Malvern Panalytical, Malvern, UK) equipped with a 405 nm laser. Samples were diluted 1:1000 in phosphate-buffered saline (PBS) before analysis. For each sample, three independent 60 s videos were recorded using the maximum detection threshold setting. Particle concentration and size distribution were subsequently calculated using NanoSight software v3.0.

### 4.4. Polar Metabolite Extraction

EV-associated metabolites were extracted using a cold methanol-based lysis and precipitation protocol. Extractions were performed starting from equivalent EV preparations normalized to 20 µg of total protein per sample determined by Bradford assay before extraction. Cold extraction solvent consisting of 80% methanol and 20% water (*v*/*v*) was added to each sample at a ratio of 4:1 (methanol:PBS, *v*/*v*). Samples were vortexed for 30 s and incubated at −80 °C for 30 min to ensure complete membrane disruption and protein precipitation. Subsequently, extracts were centrifuged at 16,000× *g* for 10 min at 4 °C. The supernatant was transferred to a new tube and dried in a vacuum concentrator. Dried extracts were reconstituted in 50 µL of acetonitrile/water (90:10, *v*/*v*), sonicated for 1 min in an ice-cold bath, and centrifuged at 16,000× *g* for 15 min at 4 °C to remove insoluble material. The resulting supernatants were collected and subjected to LC–MS-based metabolomic analysis. A pooled QC sample (prepared by combining aliquots from all samples) was injected at regular intervals to monitor instrument stability throughout the batch. Solvent blanks (acetonitrile/water, 90:10, *v*/*v*) were included to assess background signals and potential carryover. No internal standards were used. The post hoc in silico filtering of non-vesicular contaminants (e.g., apolipoproteins) was applied exclusively to the proteomic dataset. To ensure a standardized starting input across cell lines and experimental groups, metabolomic extractions were normalized based on total EV protein content. Specifically, an aliquot of the EV preparation corresponding to 20 µg of the EV protein was used as the starting material for each sample.

### 4.5. Polar Metabolome Analysis

The analysis of the polar metabolome was performed on a Vanquish Neo (Thermo Fisher Scientific, Bremen, Germany) coupled to an Orbitrap Exploris 240 mass spectrometer (Thermo Fisher Scientific). The MS was calibrated by Thermo Pierce™ FlexMix calibration solutions in both polarities. Separation was performed using a SeQuant^®^ ZIC^®^-HILIC column (150 × 2.1 mm; 3.5 µm, 100 Å) (Merck Millipore, Darmstadt, Germany). The column temperature was set at 60 °C, and the flow rate was 80 µL/min. Full MS (80–1000 *m*/*z*) and data-dependent MS/MS were performed at a resolution of 45,000 and 15,000 FWHM, respectively. The isolation window (*m*/*z*) was set to 1.6, and normalized collision energy (NCE) values of 10, 20 and 30 were used. Auxiliary gas heater temperature was 300 °C (Thermo Fisher Scientific, Bremen, Germany).

Because this study was designed for untargeted profiling and relative quantification, stable isotope-labeled standards were not utilized. Instead, analytical drift and system stability were monitored and corrected using a rigorous Quality Control (QC) strategy. Distinct pooled QC samples were prepared for each of the four cell lines by combining aliquots of extract from their respective samples (yielding 4 cell-line-specific QCs). Three biological replicates of each sample were performed for each polarity, and QC samples were randomly inserted into the batch to monitor system stability over time and facilitate run order normalization. Metabolomic analysis was conducted using Compound Discoverer software (version 3.3; Thermo Fisher Scientific), which was employed for compound detection, alignment, normalization, and identification. Compounds were grouped using an adaptive curve alignment model implemented in Compound Discoverer. Compound detection was performed using the following parameter settings: a mass tolerance of 5 ppm, retention time tolerance of 0.2 min, minimum peak intensity of 100,000 a.u., and a signal-to-noise threshold of 5. Compound identification was performed using the Human Metabolome Database (HMDB) and Massbank and Metabobase as the reference databases, and data normalization was carried out using median normalization.

### 4.6. EV Proteome Analysis

An amount of 20 µg of protein was digested with the EasyPep™ MS Sample Prep Kits (Thermo Fisher), as previously described [40]. After digestion, analysis was performed with nanoflow ultra-high-performance liquid chromatography high-resolution mass spectrometry, using a Vanquish Neo (Thermo Fisher Scientific, Bremen, Germany) coupled to an Orbitrap Exploris 240 mass spectrometer (Thermo Fisher Scientific). A volume of 1 μL of digestion was initially trapped on a PepMap trap column for 1.50 min at a flow rate of 30 μL/min (Thermo Fisher), and then peptides were loaded and separated onto a C18-reversed phase EASY-Spray™ PepMap™ Neo column (75 μm × 250 mm, 2 µm, 100 Å Thermo Fisher) with 60 min of gradient. For data-independent acquisition (DIA), a full MS scan was first acquired at a resolution of 60,000. DIA MS/MS scans were subsequently collected in the Orbitrap at a resolution of 30,000 using 8 Da isolation windows. Higher-energy collisional dissociation (HCD) was applied with a normalized collision energy of 30. DIA raw files were then processed using Spectronaut (version 20) with the DirectDIA (deep) workflow for peptide and protein identification. Carbamidomethylation was specified as a fixed modification, while protein N-terminal acetylation and methionine oxidation were set as variable modifications. A false discovery rate (FDR) threshold of <1% (q value < 0.01) was applied at both the peptide and protein levels. Proteins were considered confidently identified when supported by at least one unique peptide. Relative label-free quantification was performed based on peptide ion intensities matched to the spectral library. Data were normalized across runs using Spectronaut’s built-in automatic normalization algorithm. To account for the inherent limitations of the polymer-based EV isolation protocol, known non-vesicular contaminants that may co-purify with EVs, specifically apolipoproteins, were manually curated and excluded from the final protein dataset prior to downstream statistical and bioinformatic analyses. The complete list of excluded contaminant proteins is provided in Supplementary File S2. Subsequent pathway enrichment and network integration analyses were performed exclusively on this curated EV-enriched dataset. The mass spectrometry proteomics data were deposited in the ProteomeXchange Consortium via the PRIDE partner repository with the dataset identifier PXD081461.

### 4.7. Western Blotting

A Western blot analysis of EV preparations was performed as follows [41]. Briefly, 20 µg of total protein was separated on a 4–20% precast polyacrylamide gel and transferred onto a nitrocellulose membrane. The membrane was blocked with 5% non-fat dry milk in TBS-Tween 20 (TBS-T) and incubated overnight at 4 °C with a primary antibody against CD9 (rabbit polyclonal 1:800), CD63 (rabbit polyclonal 1:800), HSC70 (rabbit polyclonal 1:1000), LDHA (rabbit polyclonal, 1:800; Sigma-Aldrich, St. Louis, MO, USA; Merck KGaA, Darmstadt, Germany), and CALX (rabbit polyclonal 1:800; Thermo Fisher; Waltham, MA, USA). After washing, the membranes were incubated with a horseradish peroxidase (HRP)-conjugated secondary antibody (anti-rabbit IgG, 1:3000; Sigma-Aldrich, St. Louis, MO, USA; Merck KGaA, Darmstadt, Germany). Protein bands were detected using the SuperSignal™ West Pico chemiluminescent substrate (Thermo Fisher Scientific, Waltham, MA, USA). For densitometric quantification, LDHA band intensities were quantified and normalized to total protein loading, determined by the SYPRO Ruby staining of a duplicate gel run in parallel and loaded with the same samples.

### 4.8. Statistical Analysis

Statistical and multivariate analyses were performed using MetaboAnalyst 6.0. Differential abundance analyses were conducted by comparing metastatic vs. non-metastatic cell lines within each EC subtype: AN3CA vs. ISHIKAWA (Type I) and KLE vs. HEC1A (Type II). To ensure statistical consistency across the multi-omics datasets, *p*-values for both proteomic and metabolomic data were adjusted using the Benjamini–Hochberg procedure to control the false discovery rate (FDR), and q < 0.05 was considered statistically significant. Given the limited sample size typical of EV studies and the exploratory, hypothesis-generating nature of the analysis, a dual-tier statistical strategy was applied. In addition to the FDR-adjusted results, a broader exploratory set of proteins and metabolites meeting a fold change threshold of ≥1.5 or ≤0.66 together with a nominal unpaired Student’s *t*-test (*p* < 0.05) was considered. This exploratory signature was used to prioritize functionally relevant features for downstream pathway enrichment and cross-omics network integration.

Multivariate patterns were explored using PCA and PLS-DA, and hierarchical clustering was visualized using heatmaps.

### 4.9. Multi-Omics Enrichment Analysis

For multi-omics integration, the PANTHER classification system was used to identify and classify enzymes from the proteomic dataset. Pathway enrichment and enzyme–metabolite association analyses were performed using MetaboAnalyst 6.0. The network-based integration and visualization of enzyme–metabolite interactions were conducted using OmicsNet. These tools were used to identify biologically relevant pathways and functional relationships between differentially abundant proteins and metabolites.

## 5. Conclusions

This study provides an integrated metabolomic and proteomic characterization of EVs derived from Type I and Type II EC cell lines, revealing coordinated alterations in glycolysis, central carbon metabolism, purine metabolism, and inflammatory signaling. Multi-omics integration identified interconnected enzyme–metabolite networks, supporting the metabolic plasticity of EVs and highlighting their potential role in tumor progression and microenvironmental remodeling. These findings demonstrate the value of combining metabolomics and proteomics to reveal biologically relevant pathways that cannot be identified by either approach alone. Future studies should validate these signatures in patient-derived EVs and further investigate their functional and potential therapeutic targets in EC.

## Data Availability

The original contributions presented in this study are included in the article/Supplementary Materials. Further inquiries can be directed to the corresponding author.

## Supplementary Materials

The following supporting information can be downloaded at https://www.mdpi.com/article/10.3390/ijms27177703/s1.
