# Supplementary material for: Enzyme–Metabolite Network Analysis of Endometrial Cancer-Derived Extracellular Vesicles Through Integrated Proteomics and Metabolomics
Source: Int J Mol Sci. 2026 Aug 28;27(17):7703. doi: 10.3390/ijms27177703 (PMC13566025; doi:10.3390/ijms27177703)
Supplement: Supplementary file 1 [file ijms-27-07703-s001.zip › Supplementary figure S1.pdf]

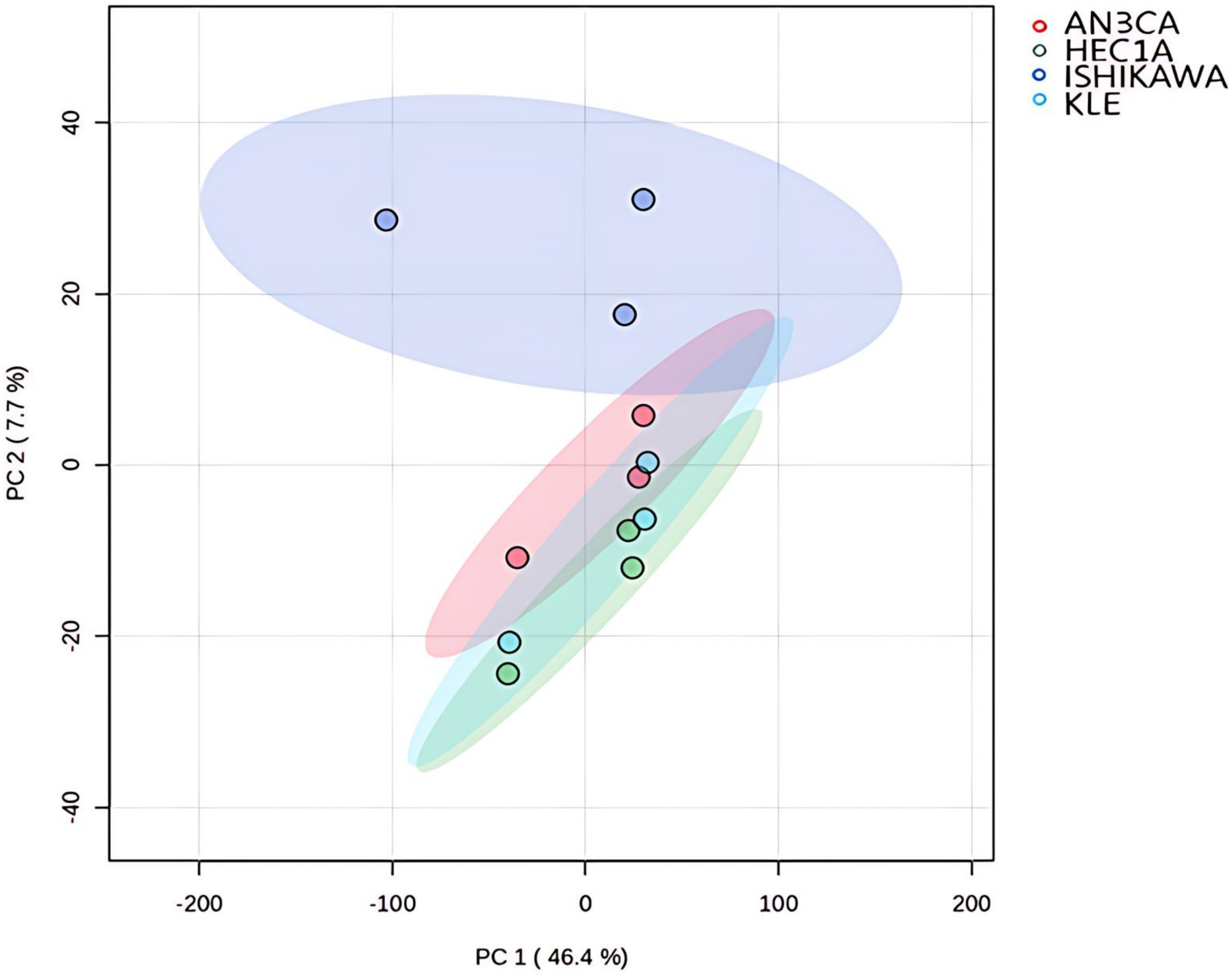


**Supplementary Figure S1.** PCA score plot of ESI+ metabolomics data showing limited separation and substantial overlap among EV samples from AN3CA, HEC1A, ISHIKAWA and KLE cell lines.
