# Supplementary material for: Enzyme–Metabolite Network Analysis of Endometrial Cancer-Derived Extracellular Vesicles Through Integrated Proteomics and Metabolomics
Source: Int J Mol Sci. 2026 Aug 28;27(17):7703. doi: 10.3390/ijms27177703 (PMC13566025; doi:10.3390/ijms27177703)
Supplement: Supplementary file 1 [file ijms-27-07703-s001.zip › Supplementary figure S2.pdf]

Figure S2. Whole membrane stained with red ponceau and Sypro Ruby

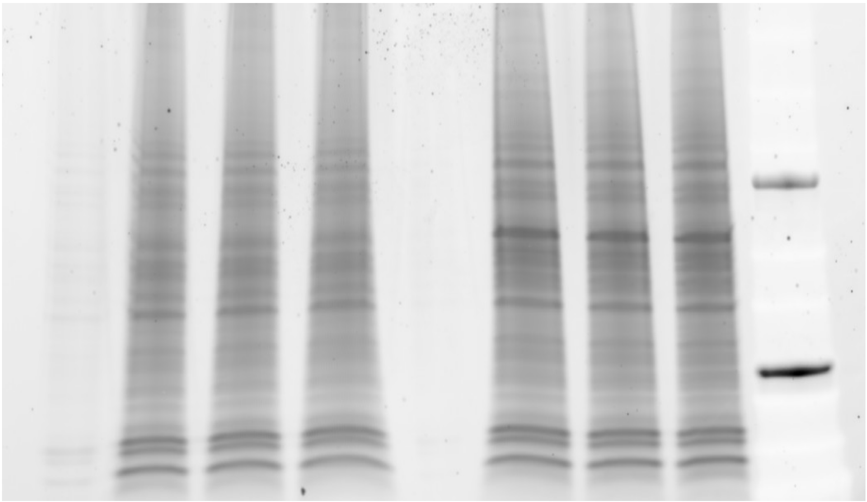

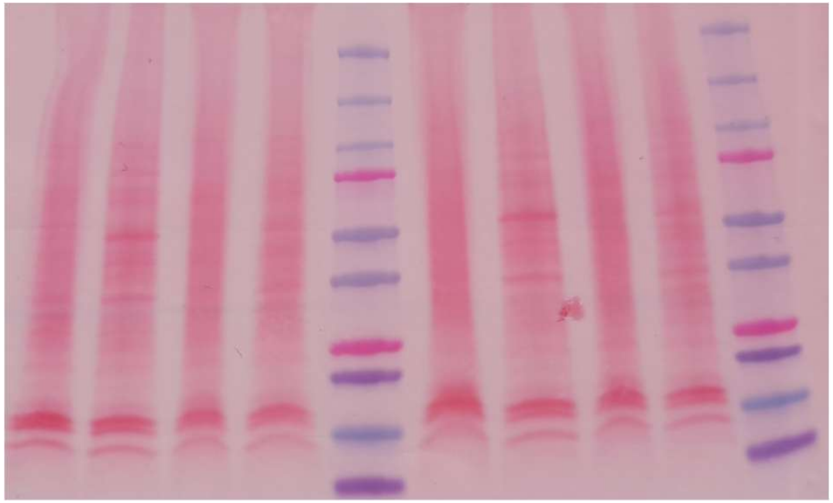

Whole membrane of the LDHA protein

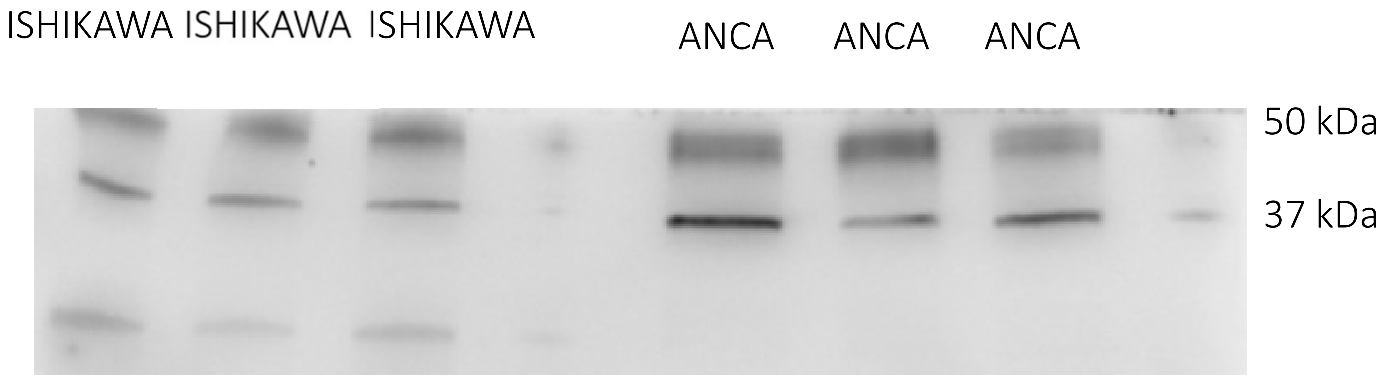

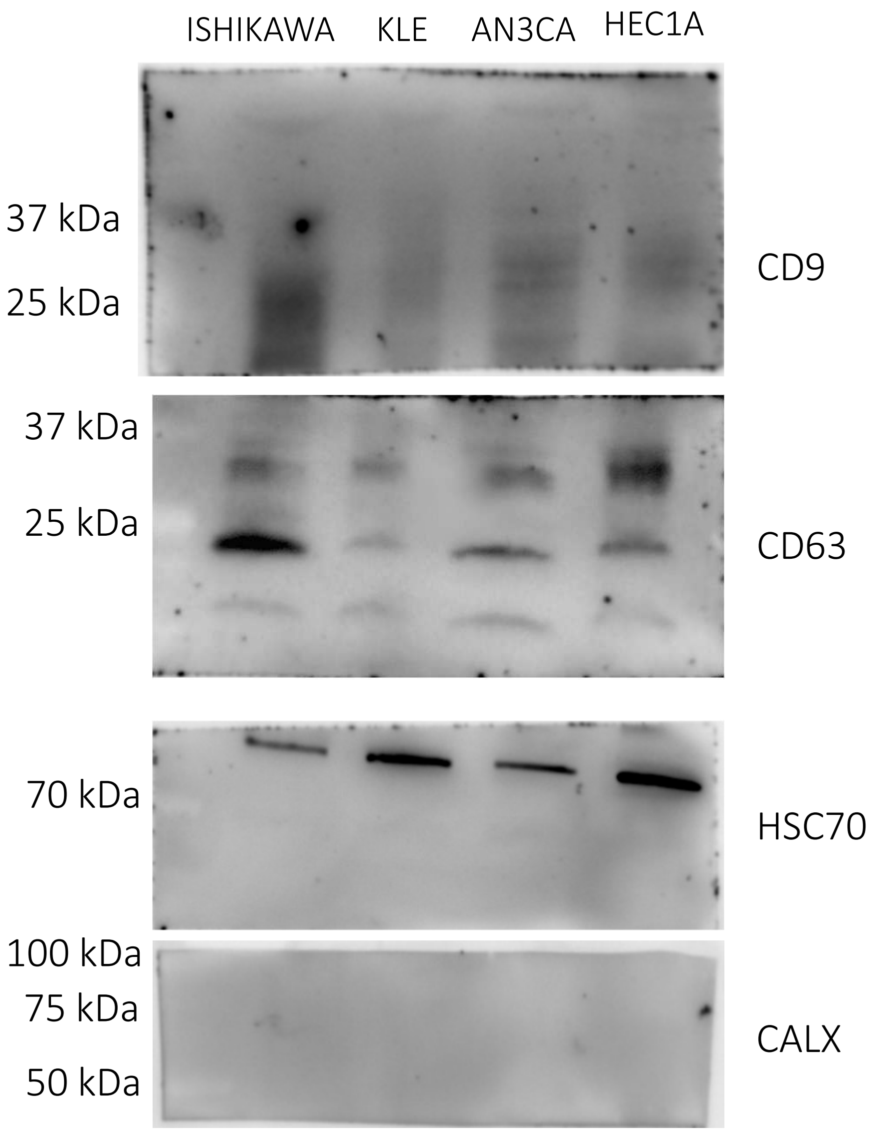
ISHIKAWA
KLE
AN3CA
HEC1A
37 kDa
25 kDa
CD9
37 kDa
25 kDa
CD63
70 kDa
HSC70
100 kDa
75 kDa
50 kDa
CALX
